# Raw and pre-processed cruise passengers' GPS tracking datasets

**DOI:** 10.1016/j.dib.2025.112078

**Published:** 2025-09-18

**Authors:** Mauro Ferrante, Andrea Perri, Stefano De Cantis, Amit Birenboim, Noam Shoval

**Affiliations:** a Department of Culture and Society, University of Palermo. Viale delle Scienze – Ed. 15, 90128 Palermo Italy; b Institute of Translational Pharmacology (IFT), National Research Council (CNR), and Department of Earth and Marine Sciences (DiSTeM), University of Palermo (UNIPA), Palermo, Italy; c Department of Economics, Business and Statistics, University of Palermo. Viale delle Scienze – Ed. 13, 90128 Palermo Italy; d Department of Geography, Hebrew University of Jerusalem, Jerusalem 91905 Israel

**Keywords:** Tracking technology, Global positioning system (GPS), Tourist mobility, Pre-Processing

## Abstract

The Global Positioning System (GPS) enables the precise collection of spatio-temporal data in real time, significantly enhancing our understanding of human mobility. GPS tracking data are spatially and temporally precise and can be supplemented using other sources. In ``Cruise passengers' behavior at the destination: Investigation using GPS technology'' by De Cantis et al. (2016) the application of this technology is exemplified to gather insightful information on spatio-temporal behaviour of cruise passengers at their destination. The study was the first to use GPS technology for analyzing the cruise tourism segment, setting a precedent in the field. Selected cruise ship passengers participated in a survey by completing initial and final questionnaires and carrying GPS data loggers during their visit. These loggers recorded geographic coordinates (latitude and longitude) along with timestamps at about ten-second intervals.

The passengers were selected using a pseudo-systematic sampling strategy, where about one out of every twenty passengers were sampled during the specified survey period. Beyond simply presenting the raw GPS data, this article also offers pre-processed data. A specially designed algorithm was employed to eliminate outliers and noise points and to impute missing values by mean of dynamic moving medians. This algorithm detects and imputes noise points in GPS data by considering both temporal and spatial distances, effectively identifying abnormal observations caused by equipment failures or environmental interference. Its efficacy was demonstrated through tests conducted with data on cruise passengers’ behavior in Palermo city (Italy).

Despite these advancements, processing GPS data for the study of tourism phenomena remains challenging. There are numerous potential metrics derivable from such data, which are crucial for understanding tourist behavior at destinations. Making these data freely available represents a significant contribution to the collaborative development of pre-processing methodologies and GPS data analysis techniques for the analysis of tourist behavior, and of human mobility in general.

The dataset holds high value for comprehending human mobility patterns and can be applied across various fields, including urban planning, transportation management, and tourism research. By systematically sampling and recording geographic coordinates along with timestamps, the dataset provides a robust foundation for the analysis of tourist mobility.

Specifications Table

**Table:** 

Subject	*Data Mining and Statistical Analysis*
Specific subject area	*Tourist mobility patterns through GPS tracking, pre-processing, and analysis of cruise passengers’ behaviors.*
Type of data	*Table, CSV files, Raw data, Pre-processed data*
Data collection	*Data were acquired using Globalsat DG-200 GPS tracking devices. Data were pre-processed using Python functions for outlier detection and imputation based on spatio-temporal relationships. The raw data were collected at about 10-second intervals and stored in CSV format. Pre-processing included removal of noise points and imputation of missing data using dynamic rolling medians and interquartile ranges.*
Data source location	*Palermo, Italy* *Latitude: 38.1157* *Longitude: 13.3615*
Data accessibility	Repository name: GPS Tracking Dataset of Cruise Passengers in Palermo City, Italy Data identification number: *Ferrante, Mauro; Perri, Andrea; De Cantis, Stefano; Birenboim, Amit; Shoval, Noam (*2024*), “GPS Tracking Dataset of Cruise Passengers in Palermo City, Italy”, Mendeley Data, V2, doi:*10.17632/7ptysvs4fs.2 Direct URL to data: https://data.mendeley.com/datasets/7ptysvs4fs/2
Related research article	De Cantis, S., Ferrante, M., Kahani, A., & Shoval, N. [1]. Cruise passengers' behavior at the destination: Investigation using GPS technology. *Tourism Management*, 52, 133–150.

## 1 Value of Data

• Making this dataset available will enable the scientific community to develop effective outlier detection techniques, crucial for maintaining the accuracy and reliability of GPS tracking data. Outliers can significantly skew analysis results, potentially leading to incorrect scientific conclusions. Addressing these anomalies ensures that the data reflects true movement patterns and behaviors. Providing this dataset will establish a solid foundation for valid scientific findings and facilitate benchmarking of new data analysis techniques.
• The data allows developing imputation techniques which fill gaps caused by signal loss or device malfunction, ensuring dataset completeness for robust scientific analysis. Accurate imputation maintains the continuity of itinerary trajectories, which is crucial for understanding tourist behaviors and mobility patterns. This allows researchers to perform comprehensive studies, develop benchmarks, and refine imputation methods for better data handling in future research.
• Advanced applications of this dataset include extracting relevant information such as identifying different activities (e.g., mode of transportation used, attraction visited) and detecting changes in mobility patterns provides researchers with deep insights into tourist behavior and mobility. This information is valuable for understanding how tourists interact with their destination and how mobility behaviors evolve over time. By identifying activities and changes, researchers can develop benchmarks for studying human mobility and test new methods for activity recognition and change detection in GPS data.
• The data provided contain detailed information about tourist behavior which can be used for cluster identification at individual and aggregate levels. Identifying clusters in GPS tracking data helps researchers understand common mobility behaviors and patterns at both individual and aggregate levels. At the individual level, this reveals personal habits and preferences, while at the aggregate level, it highlights broader trends across tourist populations. This information is crucial for researchers aiming to understand tourist behavior patterns, develop benchmarks for tourism studies, and improve clustering techniques. It also aids in solving specific problems related to the collection of information on tourist mobility (e.g. recall bias).
• The dataset permits analyzing network relationships, which may help researchers understand the connections and interactions between different nodes of tourism activity, such as attractions, restaurants, transportation, and shopping locations. At the individual level, this reveals personal trajectory and preferences, while at the aggregate level, it shows overall connectivity and flow within a destination. This analysis is essential for researchers studying tourist behavior and network dynamics, providing a common ground for comparing methods and developing benchmarks for network analysis among various points of interest within a destination.

## 2 Background

This dataset aims to enhance understanding of cruise passengers' behaviors at their destinations, providing essential information for destination management and policy-making. Utilizing GPS technology, it tracks cruise passengers' movements in Palermo city (Italy), capturing detailed data on visits to specific attractions, stay durations, and transportation modes. This data, combined with traditional methods like questionnaires, enables a comprehensive analysis of passengers' profiles and behaviors within the destination context. It addresses traditional data collection limitations, such as tourists' perceptions and recall biases, offering accurate and unbiased information about cruise passengers’ activities. The resulting data may support strategic decisions, such as: activity location choice as well as policy effectiveness evaluation.

Key studies have advanced understanding of cruise passengers’ behavior. A number of papers used GPS data to analyze spatial and temporal behavior of cruise passengers in popular mediterranean destinations, including Palermo (Italy) [1]; Dubrovnik (Croatia) [2]; Tarragona (Spain) [3]; Valencia (Spain) [4]; Copenaghen (Denmark) [5], and Gotland Island (Sweden) [6]. These studies collectively offer detailed insights into cruise passengers’ behavior and expenditure patterns, informing effective and sustainable tourism strategies.

## 3 Data Description

The dataset provides detailed information on the geographical patterns of a sample of cruise passengers in the city of Palermo (Italy), captured through GPS tracking data. These data, stored in CSV format, are individually identified, resulting in a total of 162 unique trajectories stored in the ‘Original Data’ folder. However, not all original data of the survey presented by De Cantis et al. [1] is disclosed due to homogeneity. Specifically, there were two distinct types of GPS devices in operation, each functioning differently. Thus, it was decided to provide only data recorded by means of the Globalsat DG-200 tracking device. This decision was made to ensure methodological consistency and data quality. The second device model operated using a fundamentally different logic: it entered hibernation mode when stationary and resumed data collection only upon detecting movement. This led to a discontinuous sampling profile and a statistical distribution of data points that differs markedly from those collected by the Globalsat DG-200. Moreover, each wake-up event triggered a cold start, producing clusters of spatially inaccurate points caused by delayed satellite acquisition and unstable positioning. Furthermore, only tourists who did not purchase a tour from the company and remained in the city are included in the data. Preprocessed data is also provided, organized by participant ID number in the same manner as the original data, and can be found in the folder named ‘Preprocessed Data’. A summary of the dataset’s main metrics and the corresponding trajectory IDs is presented in Table 1.

**Table 1:** Summary of the dataset's metrics, along with the corresponding IDs.

	Max Value	Max ID	Min Value	Min ID	Average Value
Number of Points Recorded	2634	P176	30	P074	1060
Trajectory Time Duration	09 h 03 m	P170	00 h 36 m	P311	03 h 58 m
Time outliers	33 %	P263	0 %	P056	15 %
Spatial outliers	93 %	P303	0 %	P005	15 %
Max distance from starting point	5.51 Km	P307	0.23 Km	P074	1.95 Km
Total distance travelled	41.14 Km	P307	0.52 Km	P074	13.9 Km
Speed	42.52 Km/h	P134	0 Km/h	P033	5.24 Km/h

Overall, the dataset not only provides a comprehensive record of passengers’ movements, but it also offers valuable insights into the diverse travel behaviors and patterns exhibited during their visit. The data are stored in a folder named **Original Data**, in which there are 161 csv files named with a unique identifier assigned to each participant, each file have the following variables:

• *Date*: The date on which the GPS data point was recorded, formatted as YYYY-MM-DD.
• *Time (local)*: The local time at which the GPS data point was captured, following the HH:MM:SS format.
• *Latitude*: Geographic coordinate (WGS1984) specifying the north-south position, expressed in decimal degrees.
• *Longitude*: Geographic coordinate (WGS1984) specifying the east-west position, expressed in decimal degrees.
• *Altitude (m)*: The vertical distance of the GPS data point above mean sea level, typically expressed in meters.
• *Speed (Km/h)*: The rate at which the object associated with the GPS data point is moving, expressed in kilometers per hour.

A separate CSV file, titled **Register**, is also provided. This file contains the start and end times for each trajectory, organized by ID. The start time refers to the moment the cruise passenger leaves the researcher's desk with the device, while the end time marks the return of the device. Points recorded before the start time are considered ``warmup points'' during which the researcher activates the device to determine its position and account for cold starts before handing it to the tourist. Points recorded after the end time capture the researcher’s movement as they return the device and download the data. Therefore, it is essential to filter the data by start and end times for each GPS trajectory csv file.

## 4 Experimental Design, Materials and Methods

In accordance with the study ``Cruise passengers' behavior at the destination: Investigation using GPS technology'' [1] tracking data of cruise passengers were acquired during the spring season 2014. Raw data provided is derived from a survey of cruise passengers disembarking in the city of Palermo between April 15 and 30, 2014. All the days in which a cruise vessel docked in the port were considered for the survey. Independent cruise passengers were selected through a pseudo-systematic sampling procedure, by selecting about one every 20 passengers disembarking from the cruise ship. All the passengers surveyed were provided with a GPS data logger device. Once the visit terminated, the passenger returned the device. Questionnaires were also administered at the survey's initiation and closure, exploring various aspects, including previous visits, socio-demographic details, attractions visited, satisfaction levels, expenditure, and income.

The resultant data, exported in CSV format, formed the foundation for an in-depth analysis of cruise passengers’ behavior at their destination. The GPS tracking data, recorded at 10-second intervals, underwent analysis using SAS 9.2 statistical software [1]. In the original study, SAS 9.2 statistical software was employed to compute basic metrics directly from the raw GPS trajectories, the preprocessing of GPS data was not systematically addressed. Although altitude and speed were recorded, they were not included in the analysis, because altitude was deemed irrelevant to the research question and the speed recorded by the device is not a precise measurement.

**Fig. 1:**
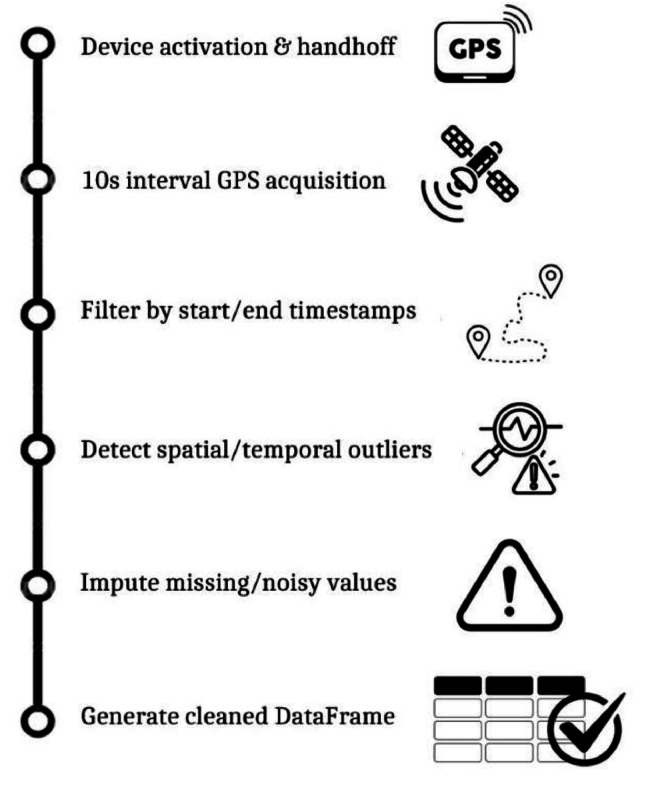
Data processing workflow.

Beyond providing raw data, pre-processed data are also provided, which have been derived by means of newly developed Python functions. These functions aim to enhance the accuracy and validation of trajectory data through a more sophisticated approach. Fig. 1 summarizes the data processing workflow.

The preprocessing of trajectory data is a critical step in ensuring data quality and reliability. The choice to adopt a statistical preprocessing pipeline based on dynamically adjusted rolling medians and IQR-based thresholds, rather than map matching techniques, was motivated by both practical and methodological considerations. First, our objective was not to reconstruct exact road-level trajectories but to correct implausible outliers and ensure trajectory continuity for behavioral analysis. Additionally, no transport mode data were collected, making it difficult to apply context-aware map matching. Finally, the computational cost of map-matching hundreds of trajectories with high-frequency data points would not yield proportional gains in trajectory interpretability for our use case. Therefore, a robust statistical approach, optimized for flexibility and generalizability, was deemed more appropriate for the study goals. The proposed methodology leverages a series of Python functions designed to handle spatial and temporal outliers effectively by means of a preprocessing pipeline.

In addition to spatial and temporal outlier detection, the preprocessing pipeline computes several trajectory-level metrics that provide analytical insight into individual movement behavior. These include:

• *Percentage of temporal outliers*, based on deviation from the rolling median of time differences (Δt);
• *Percentage of spatial outliers*, detected using rolling medians and interquartile ranges (IQRs) on haversine distance;
• *Total distance travelled*, computed as the cumulative point-to-point distance after outlier removal;
• *Maximum distance from starting point*, calculated via haversine formula;
• *Total duration and unobserved time*, defined as the time from device handoff to return minus valid observed durations;
• *Mean speed and speed percentiles*, extracted from cleaned speed records;
• *Dynamic Time Warping (DTW) distance*, measuring the similarity between raw and cleaned trajectories;
• *Rolling distance statistics and outlier intensity scores*, used during anomaly detection.

These indicators contribute to both data quality assurance and potential downstream analyses on tourist behavior.

The first function in the pipeline is the “*haversine function*”, which calculates the great circle distance in meters between two geographical points specified in decimal degrees. This calculation is essential for determining the spatial relationships between consecutive points in the dataset.

The *“dynamic_rolling_median”* function calculates the rolling median for latitude and longitude using a dynamically adjusted window size. This rolling median calculation is vital for identifying spatial outliers, as it smooths out short-term fluctuations and highlights more significant deviations from the norm. By dynamically adjusting the window size, this function ensures that the rolling median is computed over the most appropriate range of data points.

The dynamic adjustment represents a key innovation in the proposed methodology, and it is crucial because it allows the window size to adapt to the spatial distribution of the data, according to the distance between each point and the next one. The function considers the distance between points, applying a decrement factor to adjust the window size as needed.

To further enhance outlier detection, the *“dynamic_rolling_median_iqr”* function is also used. This function computes the rolling median and interquartile range (IQR) for the distance column, providing a robust statistical basis for identifying outliers. The IQR is a measure of statistical dispersion, and it is used here to determine the threshold for flagging outliers. Points that fall outside this range are flagged as potential anomalies.

The *“flag_outliers”* function applies the IQR method to flag outliers. It calculates the upper bound for each group of data points and determines whether each value exceeds this threshold. Additionally, it computes the intensity of the outliers, providing a quantitative measure of how far an outlier deviates from the expected range. This step is crucial for distinguishing between minor deviations and significant anomalies that require correction.

Once outliers are identified, the *“impute_outliers”* function is used to replace them with more accurate values. This function imputes outliers in the latitude and longitude columns using rolling median values. By replacing outlier values with interpolated ones, it is possible to maintain the integrity of the dataset while removing noise points and inaccuracies in the data.

Temporal anomalies are also addressed in the proposed methodology. The *“compute_time_difference”* function calculates the time difference between consecutive points for each trajectory. It identifies temporal outliers, which are points that have unusually long intervals compared to the median time difference. These outliers might indicate stops or signal loss, which are important factors to consider in trajectory analysis.

The *“trajectory_cleaner”* function integrates all the aforementioned steps into a cohesive data cleaning process. This function processes the trajectory data by first calculating distances between consecutive points using the haversine function. It then adjusts the window size dynamically, computes rolling medians and IQR, flags and imputes spatial outliers, and identifies temporal outliers. The result is a refined DataFrame that includes essential columns such as identifiers, coordinates, dates, times, spatial outliers, time outliers, and imputed values. How each type of point is treated is summarized in Table 2.

**Table 2:** Summary of terminology used in the preprocessing of GPS trajectory data, including definitions, detection methods, treatment strategies, and relevant algorithmic parameters.

Term	Definition	Detection Method	Treatment Strategy	Parameter Notes
**Noise point**	Raw GPS reading that deviates spatially from adjacent points, often due to cold start or signal loss	Dynamic rolling median with distance-adaptive window + IQR on haversine distances	Imputed using dynamic rolling median	base_window=13, distance_interval=10, decrement_factor=2, iqr_threshold=2
**Spatial outlier**	Point whose position sharply deviates from expected spatial trajectory	Rolling median smoothing + haversine + dynamic window size + IQR	Flagged and imputed	Sensitive to transportation mode (e.g., walking vs. bus)
**Temporal outlier**	Point recorded after an unusually large time gap from previous point	Inter-point time delta vs. rolling median + IQR	Flagged (not imputed)	Helps identify stops or GPS unavailability
**Intensity of outlier**	Quantitative score indicating degree of deviation beyond IQR threshold	Calculated as multiple of IQR distance from rolling median	Retained as metadata for each flagged point	Helps prioritize correction or model tuning

The key enhancements in this updated methodology include the dynamic adjustment of window sizes and the integration of rolling median and IQR calculations. By adapting the window size to the spatial distribution of the data, the rolling medians are computed over the most relevant data points, improving the accuracy of outlier detection. The use of IQR provides a robust statistical framework for identifying outliers, while the imputation of outliers maintains data integrity.

**Fig. 2:**
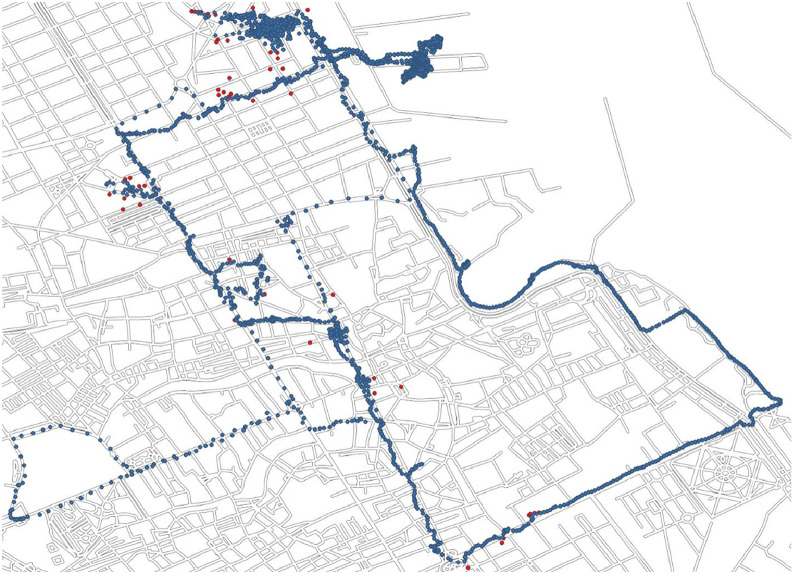
Example map of a GPS trajectory collected from a cruise passenger (ID: P002) during their visit to Palermo. Blue points represent valid GPS locations retained after preprocessing; red points indicate spatial or temporal outliers identified and removed.

To visually demonstrate the impact of the preprocessing pipeline, in Fig. 2, a representative map showing a sample trajectory before and after data cleaning is displayed. Blue points indicate retained GPS positions, while red points highlight spatial or temporal outliers identified and removed by the preprocessing algorithm. This visual illustration helps clarify how erroneous segments are corrected and noisy observations excluded to yield a reliable trajectory.

The proposed methodology offers a sophisticated approach to preprocessing trajectory data, combining advanced statistical techniques with dynamic adjustments to ensure data quality and reliability. The resulting refined dataset is well-suited for further analysis and applications in various fields.

This approach encounters challenges in effectively handling outlier values at the initial data points, often attributed to the cold start phenomenon. It refers to the situation where a GPS device has just been powered on or initialized or has no prior knowledge of its current location or the location of the GPS satellites signals and navigation data and calculate a position solution after being turned on. This can result in longer acquisition times for GPS signals and a higher chance of signal loss or interference. During this time, the GPS data may contain outlier values [7]. They are typically caused by the GPS receiver attempting to estimate its location based on incomplete or noisy data, and they tend to be spread out over time and space. In order to determine its location, a GPS device needs to receive signals from at least four GPS satellites.

The resulting DataFrame encompasses essential columns such as identifier, latitude, longitude, date, time, noise points, time outliers, and the calculated time outlier threshold. This comprehensive method ensures accurate trajectory data preprocessing, incorporating temporal and spatial outlier identification, as well as data imputation, resulting in a refined dataset.

## Limitations

The data presented in this article has several limitations. The sampling procedures were confined to April, which does not account for potential seasonal variations in passengers’ behavior. The data is also specific to a single destination, introducing a destination effect that clearly may be influenced by destination characteristics, and attractions. This affects data quality, as various destinations possess distinct characteristics that influence GPS data. Additionally, only independent cruise passengers were included, excluding those who purchased a tour through the cruise company. Of the two GPS models used in the study, only the Globalsat data is provided here for consistency, though this choice affects the type of data obtained. Signal loss issues arise from the nature of attractions (indoor vs. outdoor), transportation modes (vehicles vs. on foot), and urban canyon effects caused by street width and building height [8]. While various preprocessing methodologies are discussed in the literature [9], the one proposed here is just one of many possible approaches, dependent on the available data type. Additionally, the dataset was pre-processed without using map matching techniques. While this may limit the precision of route alignment on the street network, our statistical method effectively identifies and corrects spatial and temporal anomalies using robust, data-driven thresholds. We visually validated the cleaned trajectories and found that the pipeline preserves essential behavioral patterns (e.g., stop locations, movement segments). Future work could explore hybrid approaches that incorporate map-matching when enriched network data and transport mode labels are available.

## Ethics Statement

The data collection for the study titled ``Raw and pre-processed cruise passengers' GPS tracking datasets'' was conducted in 2014. At that time, the research adhered to the prevailing ethical standards and guidelines. Participants were informed about the nature and purpose of the study, and their consent was obtained in accordance with the ethical practices of the period. Data have been collected in an anonymous way, ensuring that no personally identifiable information is present. This approach aligns with current ethical standards for data sharing and publication, prioritizing participant confidentiality and data protection. By implementing these anonymization procedures, we mitigated any potential ethical concerns related to the use of the data in this study.

## Data Availability

Mendeley DataGPS Tracking Dataset of Cruise Passengers in Palermo City, Italy (Original data).

## References

[1] De Cantis S., Ferrante M., Kahani A., Shoval N. (2016). Cruise passengers' behavior at the destination: investigation using GPS technology. Tour. Manag..

[2] Ferrante M., De Cantis S., Shoval N. (2018). A general framework for collecting and analysing the tracking data of cruise passengers at the destination. Curr. Issues Tour..

[3] Domenech A., Gutierrez A., Clavé S.A. (2020). Built environment and urban cruise tourists' mobility. Annals Tour. Res..

[4] Casado-Díaz A.B., Navarro-Ruiz S., Nicolau J.L., Ivars-Baidal J. (2021). Expanding our understanding of cruise visitors' expenditure at destinations: the role of spatial patterns, onshore visit choice and cruise category. Tour. Manag..

[5] Sciortino C., Ferrante M., De Cantis S., Gyimóthy S. (2022). Tracking cruise passengers' consumption: an analysis of the relationships between onshore mobility and expenditure. Annals Tour. Res. Empir. Insights.

[6] Toger M., Östh J., Persson S.G. (2023). What you see is where you go: cruise tourists’ spatial consumption of destination amenities. Econ. Themes.

[7] Shoval N. (2008). Tracking technologies and urban analysis. Cities.

[8] Shen L., Stopher P.R. (2014). Review of GPS travel survey and GPS data-processing methods. Transport Rev..

[9] Abbruzzo A., Ferrante M., De Cantis S. (2021). A pre-processing and network analysis of GPS tracking data. Spat. Econ. Anal..

